# Outbreak of oral ulcers (glossitis) among the students of three central schools under Tsirang district Bhutan, 2018

**DOI:** 10.1186/s12903-021-01808-5

**Published:** 2021-09-14

**Authors:** Tshewang Gyeltshen, Lham Dorji, Leki Dorj, Kuenga Choden

**Affiliations:** 1 Tsirang Hospital, Damphu, Tsirang District Bhutan; 2 Bhutan Health and Medical Council, Thimphu, Bhutan; 3 Yebilaptsa Hospital, Zhemgang District, Bhutan

**Keywords:** Glossitis, Oral Sores, Nutritional Deficiency, Bhutan, Outbreak

## Abstract

**Background:**

Nutritional deficiencies are common worldwide and is most notable in low and middle income countries. In the early weeks of September 2018, oral sores mostly affecting the tongue were reported in three central schools under Tsirang district, Bhutan. A total of 204 students were affected in the three central schools. All the affected students have been assessed on the outbreak and nutritional survey was conducted in the three schools**.**

**Methods:**

A total of 204 students who complained of different oral lesions in the affected three schools were screened by the dental surgeon for clinical assessment and administered questionnaire for nutritional assessment by the nutritionist. Twenty-one students randomly selected were sent for blood tests for micronutrient assays and complete blood count. 41 students were tested for Exfoliative cell cytology to test for fungal elements. The collected data was double entered and validated using Epi-Data version 3.1 and analysed using Stata 15 IC. The characteristics of the participants are presented as frequencies, percentages, mean and standard deviation (SD).

**Results:**

Almost all the affected students in all the three schools had complaints of Burning Sensation (n = 158) followed by Pain (n = 153), Impaired Taste Sensation (n = 100) and Intolerance to Spicy Food (n = 95). Nutritional assessment analysis suggested schools not meeting the Recommended Dietary Allowance (RDA) for all components of Vitamin B complexes and Iron.

**Conclusion:**

Clinical assessments and nutritional survey analysis found inadequate vitamin rich dietary intakes among all the three schools. Implementation of a strict school feeding program with a balanced diet has been suggested accordingly.

## Background

Diet imbalance has detrimental effect to both physical and mental growth of a child causing systemic illnesses and death [1, 2]. Globally, nutritional deficiency affects approximately two billion people and is considered one of the major contributors to childhood morbidity and mortality [3]. Nutritional deficiency is a widespread phenomenon in developing countries mostly affecting the poor and socioeconomically disadvantaged ones [4]. Deficiencies of essential micronutrients including iron and components of vitamin B complex can manifest in oral cavity largely affecting tongue and the oral cavity mucosal linings [5].

Bhutan is a low and middle income country in South Asia. The schools in Bhutan form one of the remotest among the worlds. The school feeding program was instituted in Bhutan in 1974 with the support from the World Food Programme (WFP) [6]. The WFP upon phasing out in the year 2018, provided technical support in institution of national school feeding program and concurrently initiated the feeding of fortified rice in the schools.

Tongue lesions are a disorder of primary concern regarding oral and general health [7]. The glossitis may be caused by any of the following causative factors or in combination such as nutritional deficiency, underlying systemic diseases, microbial infections, concurrent medications and unhealthy oral habit [8]. Glossitis can be consequent to inadequate dietary intake of iron and components of vitamin B complex such as B1 (Thiamine), B2 (Riboflavin), B3 (Niacin), B6 (Biotin), B9 (Folate) and B12 (cyanocobalamin) [9].

In September 2018, clusters of oral sores mostly affecting the tongue were reported in three central schools under Tsirang district through Bhutan’s Ministry of Health, Health Surveillance Reporting system. The outbreak investigation team comprising of the district health officer, dental surgeon and the nutritionist visited the affected schools. Nutritional deficiency outbreaks among schools in Bhutan has been reported before with an outbreak of peripheral neuropathy in number of schools [10, 11].

This paper aims to describe (1) Presenting complaints of the affected students, (2) clinical manifestations of the outbreak of Glossitis and (3) the school diet assessment done using semi-quantitative food frequency questionnaire on the affected students.

## Methods

### Study design

Descriptive Study.

### Setting

Bhutan is a small landlocked country in South Asia. It is sandwiched between the northern plains of India and south of the Tibetan plateau of the People’s Republic of China. The country is divided into twenty administrative divisions called Dzongkhags (districts) with approximately 0.7 million populations. The schools in Bhutan are usually government schools with the Royal Government providing free access to education till high school level. The enrolled students are either boarding students or day students. The boarding students are provided with government supplied food from school mess derived from school feeding program under Ministry of Education throughout their stay in the school. The day school students get only lunch from the school.

The first cases of oral sores were detected among the students of Tsirangtoe Central School on 3rd September 2018 in the outpatient department of Tsirangtoe BHU. Upon interception, it was found that two other central schools in the district were affected with similar lesions. This study was conducted in the affected three schools under Tsirang district, Bhutan from 3rd September 2018 to 30th September 2018. A total of 204 affected students with diagnosed Glossitis manifesting varying degrees of tongue lesion were enrolled in the study.

The total number of students in the district at the time of this study was 2972 [12]. Tsirangtoe Central School, Damphu Central School and Mendrelgang Central School were the three schools affected with the outbreak. The schools have both boarding and day schooling facilities. In all the three schools, food is prepared by school’s cooks in a kitchen which is located separately from other school’s buildings. The boarding students are provided with the three meals and evening tea. They follow a standard food menu which contains fortified rice, pulses and mixed vegetables for lunch and dinner. Egg is provided three times a week while meat is given once weekly.

### Study population

Affected students enrolled as a full time regular student of the central schools and those manifesting oral lesions during the period of reported outbreak.

### Exclusion criteria

1. Students presenting with Odontogenic Infections.
2. Students presenting with tonsillar and peritonsillar Inflammations.
3. Students with Non Mucosal oral lesions
4. Students with Mucosal Lesions other than the tongue.
5. Students who were affected but absent on the day of investigation

### Case diagnosis

Glossitis as a lesion of the tongue was suspected clinically when any of the symptoms such as pain, swelling, burning sensation, intolerance to spicy food, impaired taste sensation, depapillation and inflammation was noted. Any of these symptoms as found in the enrolled subjects, manifested alone or in combination constituted a form of glossitis and thus were included in the study.

### Nutritional analysis and dietary assessment

Nutrition and dietary assessment were carried out among the affected schools with the use of in interviewer administered Typical Day 24 Hours Food Recall (24HR) on the day of data collection and analysed using Nutrisurvey2007 software. A structured questionnaire as prescribed in Food and Agriculture Organization of the United Nations has been used for the purpose. All the affected students, both boarder and days scholars were interviewed with the use of 24-HR dietary recall questionnaire. The school kitchen and the scheduled school food menu were also assessed.

### Terms and definitions

1. **Vegetarian**: In context on this study, vegetarians were students who did not take any form of meats, fish or eggs; however, dairy products in the forms on milk, cheese or curds were being taken.

### Sampling

The study included all those affected students meeting the inclusion criteria.

### Ethics approval and consent to participate

Ethics approval has been approved by the Research Ethics Board of Health (REBH), Ministry of Health, Royal Government of Bhutan vide approval number *Ref. No. REBH/Approval/2019/067.* An informed consent has been obtained from the individual participants for the use of photographic materials while the use of data and consent to participate had been obtained from the legal guardian (Principal of the school). All methods were carried out in accordance with relevant guidelines and regulations as enshrined in Helsinki Declarations 1964.

### Data and variables

#### Sociodemographic variables

Sociodemographic variables such as Name, Age, gender, whether boarding or day-scholar, dietary habit and grade at which they studied were obtained from all the affected students using a pre-designed data collection form by the author.

#### Clinical variables

Clinical Variables such as Body Mass Index (BMI), presenting complaints, and clinical manifestations of tongue were collected from all the affected students using a pre-designed data collection form by the author.

#### Laboratory investigations

Laboratory Investigations on Serum Vitamin B Complex Assay, Complete Blood Counts and Exfoliative Cell Cytology for Fungal Elements have been tested on selected students. Due to limited resources and having had to ship the samples to another centre for testing, only 21 (for serology testing & Complete Blood Counts (CBC)) and 40 (for cell cytology testing) out of 204 were selected. Employing systematic random sampling; a student at every 10th serial was recruited for the serology and CBC testing; and a student at every 5th interval was recruited for Exfoliative cell cytology testing. A total of 21 blood samples; 13 from Tsirangtoe Central School and 8 from Damphu Central School had been shipped to Royal Center for Disease Control (RCDC) public Health lab for Serology Testing for Vitamin B Complex Assay. The Exfoliative Cell Cytology and the CBC were conducted in Tsirang Hospital.

### Data management and analyses

The data was entered and managed using Epidata Entry Software version 3.1 (Version 3.1, EpiData Asdsociation, Odense, Denmark). A double entry was made and validated. Data analysis was carried out using Stata 15 IC (StataCorp. 2017. *Stata Statistical Software: Release 15*. College Station, TX: StataCorp LLC).

Analysis included summarization of clinical complaints and case presentations as seen on the day of outbreak investigation. Descriptive statistics commands such as frequencies, percentages, mean, median and standard deviation were used to describe the study variables. Nutrition and dietary assessment were analysed using the Nutrisurvey2007 software.

## Results

Of 2972 students in three central schools, 276 students were screened exhibiting various complaints. Of these 204 students (6.9%) were identified to have been affected by the outbreak. 72 students exhibiting mostly odontogenic, tonsillar and peritonsilar inflammatory complaints were excluded from the study. Of the total students, 2 students were reported to be absent on the day of investigation. Among the total of 204 students being affected, 104 (51%) were male student and 100 (49%) were female. Majority of the affected students were boarding students accounting to 89% (n = 182) of the affected students. 22 (11%) of the affected students were day schoolers and were getting only a daytime meal from the schools. Most of the students are non-vegetarian with only 30 (14.7%) students reported to be vegetarian. Other characteristics of the affected students are described in Table 1.

**Table 1:** Characteristics of the affected students in three central schools; Tsirang, 2018

	SCHOOLS (N = 204)			
	Tsirangtoe (n = 74)	Damphu (n = 72)	Mendrelgang (n = 58)	TOTAL (n = 204)
*Age (Years)*				
< 10	17	0	3	20 (9.8%)
10–15	52	5	37	94 (46.1%)
≥ 15	5	67	18	90 (44.1%)
*Gender*				
Male	23	45	36	104 (51.1%)
Female	51	27	22	100 (49.9%)
*Boarder/Day-Scholar*				
Boarder	62	68	52	182 (89.2%)
Day-Scholar	12	4	6	22 (10.8%)
*Dietary Habit*				
Non Vegetarian	61	63	50	174 (85.3%)
Vegetarian	13	9	8	30 (14.7%)
*Classes*				
Class ≤ VI	31	0	11	42 (20.6%)
Classes VII-IX	43	10	44	97 (47.5%)
Classes ≥ IX	0	62	3	65 (31.8%)
*BMI (kg/m*^*2*^*)*				
Underweight	41	10	13	64 (31.4%)
Normal	30	60	44	134 (65.6%)
Overweight	2	1	1	4 (1.8%)
Obese	1	1	0	2 (1.2%)

**Fig. 1:**
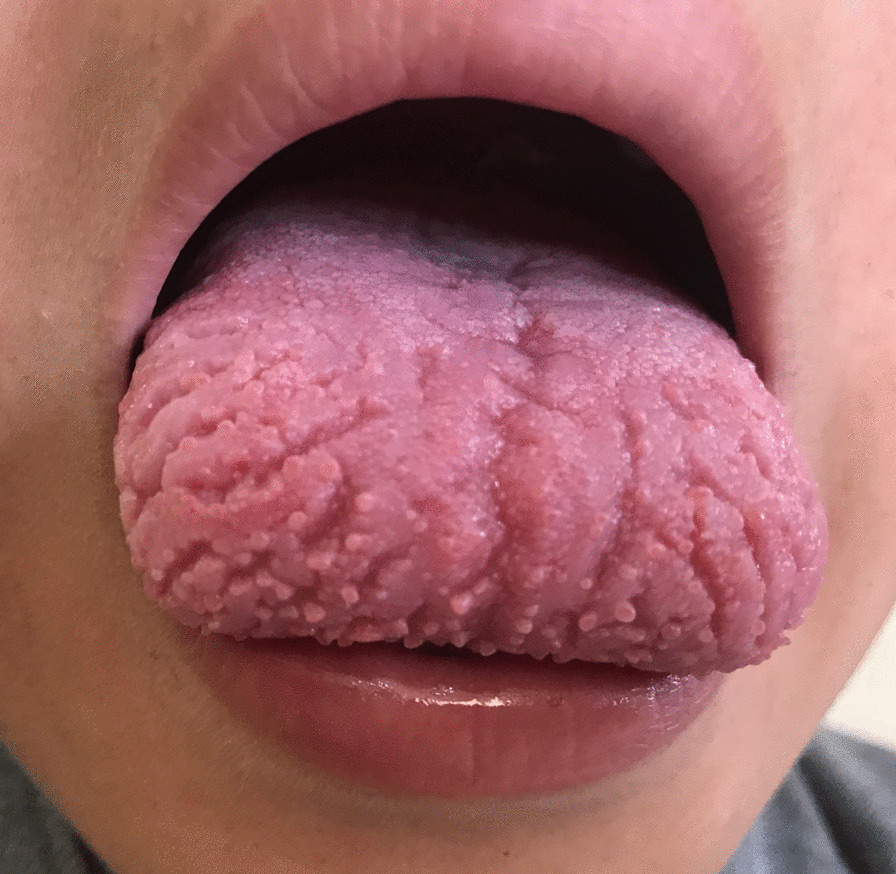
Fissured tongue (lingua plicata)

**Fig. 2:**
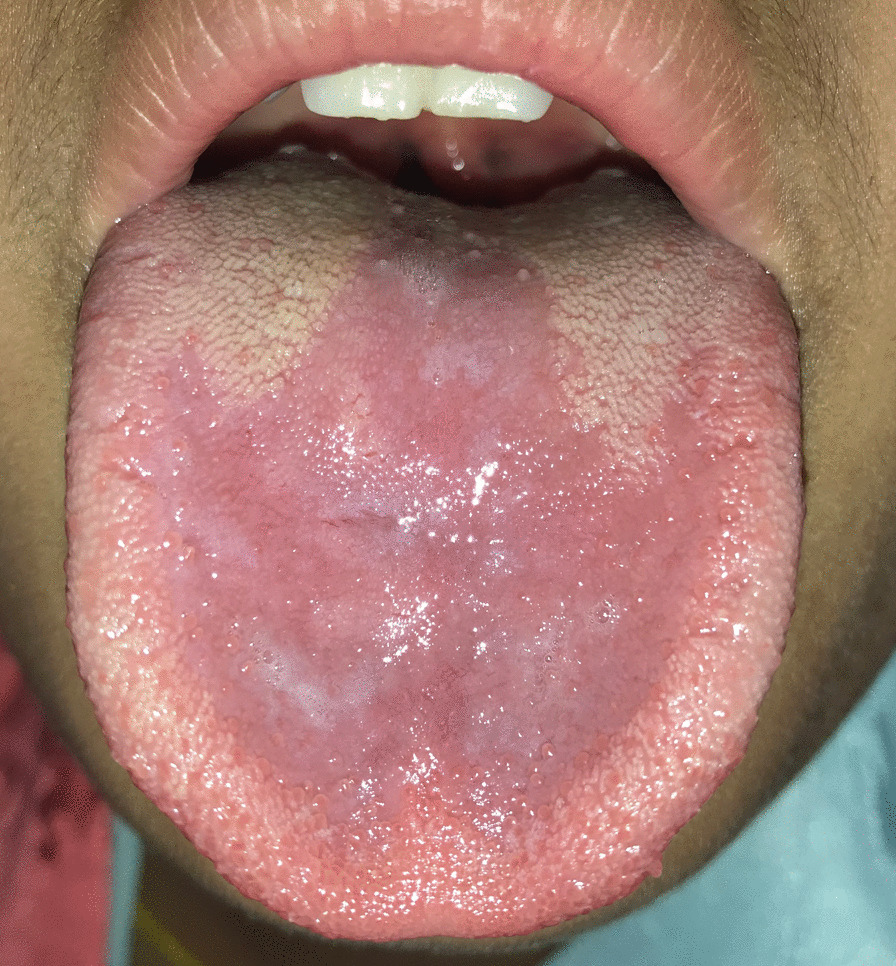
Atrophic glossitis

**Fig. 3:**
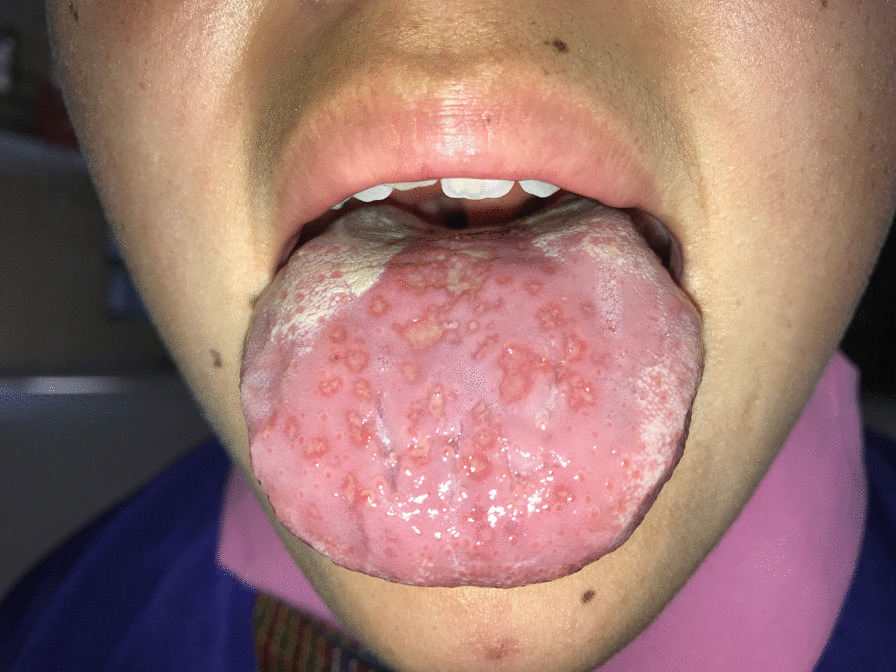
Glossitis with secondary infections

Almost all the affected students had complaints of Burning Sensation of the tongue followed by Pain, Impaired Taste Sensation and depapillation of the tongue as described in Table 2. The site most affected was the dorsum and anterior 2/3rd of the tongue. Analysis on clinical assessment found out that students were suffering largely from Fissured Tongue (Fig. 1) followed by Atrophic Glossitis (Fig. 2) and Allergic Stomatitis. Some of the students also exhibited secondary opportunistic infections (Fig. 3).

**Table 2:** Clinical presentation of the affected students in three central schools; Tsirang, 2018

	STUDENTS (N = 204)
*Presenting complaints*	
Burning Sensation	158 (77.0%)
Pain	153 (75.0%)
Impaired Taste	100 (49.0%)
Intolerance to spicy food	95 (47.0%)
Fissured Tongue/Depapillated	34 (16.6%)
*Clinical presentation*	
Fissured Tongue	172 (84.3%)
Depapillated Tongue	147 (72.1%)
Allergic Stomatitis	42 (20.6%)
Median Rhomboid Glossitis	35 (17.2%)
Foliate Papillitis	30 (14.7%)
Oral Candidiasis	26 (12.7%)
Recurrent Apthous Stomatitis	11 (5.4%)
Others	13 (6.3%)

**Fig. 4:**
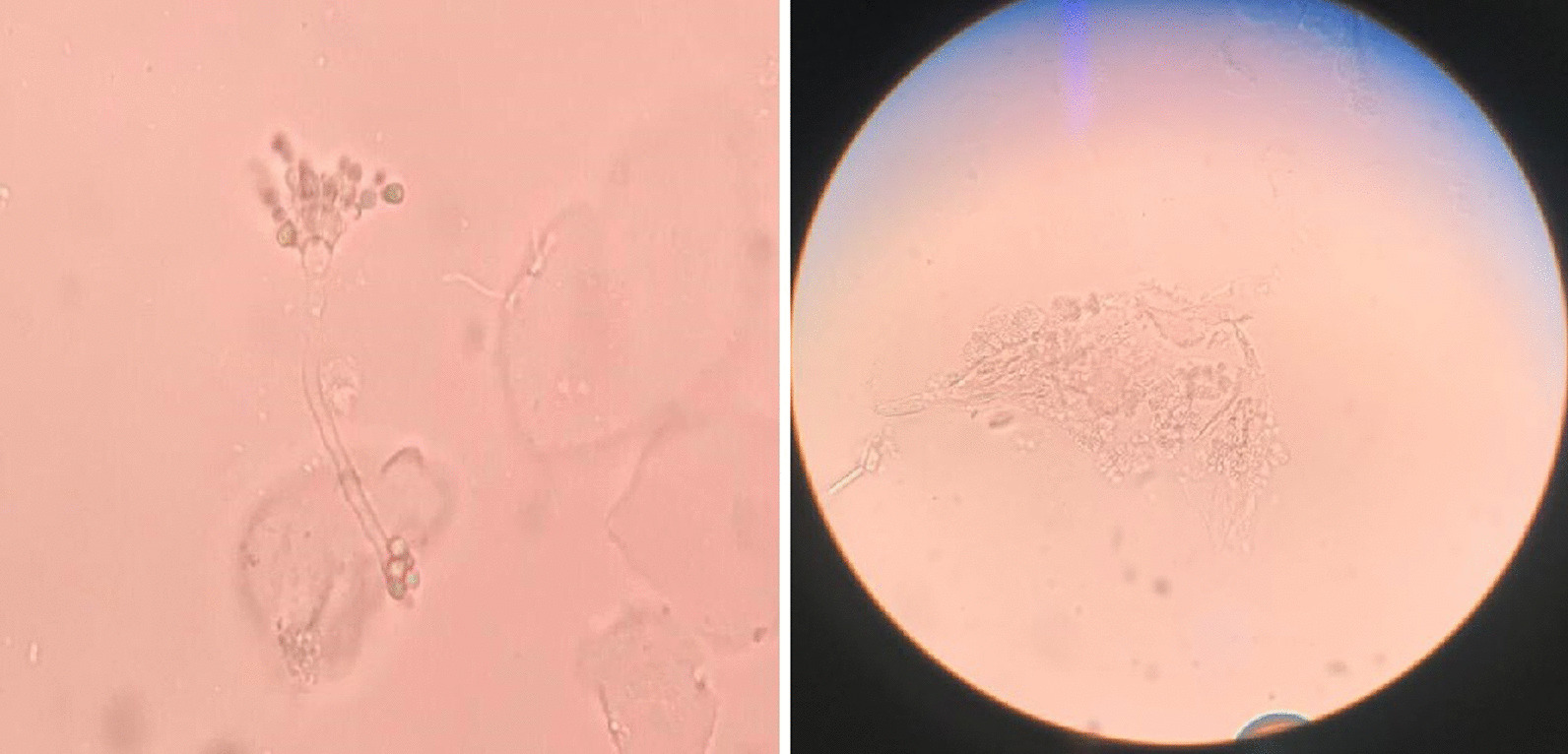
Fungal elements as seen under the microscope

The laboratory blood investigations for haemoglobin and the red cell indices were all in within the normal ranges as described in Table 3. The vitamin B complex assay on all the tested sample yielded normal levels for Vitamins B1, B6, and B12. However, vitamins B2 and B9 were reported below normal range specified by the kit manufacturer as mentioned in Table 4. Of the 40 cases randomly accrued for Exfoliative Cell Cytology Testing to see fungal elements for candida species, 31 students were tested positive for the fungal elements (Fig. 4).

**Table 3:** Median test results of vitamin B complex assays for 21 affected students in Tsirang, 2018 (n = 21)

Parameter tested (nmol/L)	Test results (median)	Range (nmol/L)
VitB1	286.1	234 to 290
VitB2	***102***	***106.5–638.5***
VitB6	247.8	194.8 to 290.2
vitB9	***3.2***	***4.5–45.3***
vitB12	685.1	298.8–1092.0

The bolditalics was to stress that these test result values are lower than normal range for the given test

**Table 4:** Red cell indices for 21 affected students in Tsirang, 2018 (n = 21)

Parameter tested	Test results Mean ± SD	Range/total (N = 21)
Red Blood Cell (10^6^/ul)	4.56 ± 0.26	3.8–5.2
Hematocrit (%)	41 ± 1.60	33.0–43.0
MCH (pg)	34.02 ± 1.35	27.0–36.0
*MCV (fl)*	75.30 ± 2.37	73.0–88.0
Microcytic		3
Normocytic		18
Macrocytic		0
*MCHC (g/dL)*	34.75 ± 0.54	30.0–38.0
Hypochromic		2
Normochromic		19
Hyperchromic		0
*Haemblogbin (g/dL)*	13.20 ± 2.61	11.0–16.0
Anemic		4
Non-anemic		17

Nutritional Survey analysis found inadequate vitamin rich dietary intakes among all the three schools. All three schools' diet menus were lacking of green leafy vegetables as found from the scheduled kitchen menus. From the Typical Day 24-Hour Recall from the cases, it was found that the diet on that particular Day didn’t meet Recommended Dietary Allowance (RDA) for all the vitamin B complexes and Iron as described in Table 5 [13, 14].

**Table 5:** Typical day 24-hour recall analysis for affected students in Tsirang, 2018

Vitamins	Mean ± SD	Recommended Dietary Allowances (RDA) Range
VitB1, (Thiamine) (mg/d)	0.88 ± 0.12	0.8–1.5
VitB2, (Riboflavin) (mg/d)	0.25 ± 0.34	1.0–1.8
VitB3(Niacin) (mg/d)	11.69 ± 2.50	13–17
VitB6 (Pyridoxine) (mg/d)	1.03 ± 1.43	1.6–2
VitB9 (Folic acid) (mcg/d)	95.56 ± 2.36	120–200
VitB12 (cyanocobalamin) (mg/d)	0.20 ± 0.12	0.2–1
Iron (mg/d)	8.59 ± 1.82	21–26

## Discussion

This is the first reported outbreak of Glossitis among the students within Bhutan with none having reported before. Of 204 students affected with the glossitis, most of them exhibited fissured and depapillated tongue. The laboratory analysis showed low median values for vitamins B2 and vitamins B9. The nutritional analysis is suggestive of low intake of vitamins and mineral rich containing foods in all the affected three central schools.

Iron and Vitamin B complex constituents notably B9 (Folate) and B12 (Cyanocobalamin) are among the important nutritional components that affect oral and general health [15]. Various oral symptoms such as glossitis, glossodynia, Recurrent Aphthous Ulcers, cheilitis, dysgeusia, lingual paresthesia, burning sensations and pruritus have been reported among individuals having decreased levels of Iron and vitamin B12 intake [15, 16].

The low median values for vitamins B2 and B9 are indicative of poor diets in schools. This is further supported by the nutritional analysis which shows low intakes of all vitamin containing foods in the schools. Previous studies conducted among the boarding schools in Bhutan reported the lack of thiamine and cobalamin among the students [17, 18]. The findings from this study suggests similar underlying nutritional deficiency in affected schools.

## Limitations of the study

This study is carried out ad hoc in response to the outbreak investigation as reported in the ministry of health’s disease surveillance reporting system. Due to limited resources only limited number of laboratory investigations were administered. We could not conduct other important laboratory investigations which could have been valuable to this study such as peripheral blood smear (PBS), Total Iron Binding capacity (TIBC) and transferrin saturations (TSAT) due to lack of facility in the local primary healthcare centre. The local health assistants have supplemented some of the students who visited the local primary health centres for oral sore complaints with the vitamin B complex and as such may have affected our serum analysis report. However, this study generally establishes the underlying problems of other schools across the country.

## Conclusions

The clinical assessments and the nutritional survey analysis on affected students found inadequate vitamin rich dietary intakes among all the three schools. Implementation of a strict school feeding program with a balanced diet has been suggested.

## Data Availability

Data and materials available with the corresponding author upon request.

## Abbreviations

RDA: Recommended Dietary Allowance
SD: Standard Deviation
WFP: World Food Programme
REBH: Research Ethics Board of Health, Ministry of Health, Bhutan
BMI: Body Mass Index
CBC: Complete Blood Count
RCDC: Royal Centre for Disease Control
TCS: Tsirangtoe Central School
DCS: Damphu Central School
MCS: Mendrelgang Central School
